# Gene expression of axenically-isolated clinical *Entamoeba histolytica* strains and its impact on disease severity of amebiasis

**DOI:** 10.1371/journal.ppat.1010880

**Published:** 2022-09-30

**Authors:** Yasuaki Yanagawa, Shinji Izumiyama, Yumiko Saito-Nakano, Kumiko Nakada-Tsukui, Seiki Kobayashi, Naoko Yoshida, Yoshimi Kikuchi, Hiroyuki Gatanaga, Shinichi Oka, Tomoyoshi Nozaki, Koji Watanabe

**Affiliations:** 1 AIDS Clinical Center, National Center for Global Health and Medicine, Tokyo, Japan; 2 Department of Parasitology, National Institutes of Infectious Diseases, Tokyo, Japan; 3 Department of Infectious Diseases, Keio University School of Medicine, Tokyo, Japan; 4 Department of Tropical Medicine and Parasitology, Juntendo University School of Medicine, Tokyo, Japan; 5 Joint Research Center for Human Retrovirus Infection, Kumamoto University, Kumamoto, Japan; 6 Department of Biochemical Chemistry, Graduate School of Medicine, The University of Tokyo, Tokyo, Japan; Jr., University of Virginia, UNITED STATES

## Abstract

The severity of *Entamoeba histolytica* infection is determined by host immunology, pathogen virulence, and the intestinal environment. Conventional research for assessing pathogen virulence has been mainly performed using laboratory strains, such as a virulent HM-1: IMSS (HM-1) and an avirulent Rahman, under various artificial environmental conditions because of the difficulties of axenic isolation of the clinical strains. However, it is still unclear whether scientific knowledge based on laboratory strains are universally applicable to the true pathogenesis. Hereby, we performed transcriptomic analysis of clinical strains from patients with different degrees of disease severity, as well as HM-1 under different conditions. Even after several months of axenization, Clinical strains show the distinct profile in gene expression during in vitro passage, moreover, difference between any 2 of these strains was much greater than the changes on the liver challenge. Interestingly, 26 DEGs, which were closely related to the biological functions, were oppositely up- or down regulated between virulent Ax 19 (liver abscess) and avirulent Ax 11 (asymptomatic carrier). Additionally, RNAseq using laboratory strain (HM1) showed more than half of genes were differently expressed between continuously in vitro passaged HM1 (in vitro HM1) and periodically liver passaged HM1 (virulent HM1), which was much greater than the changes on the liver passage of virulent HM1. Also, transcriptomic analysis of a laboratory strain revealed that continuous environmental stress enhances its virulence via a shift in its gene expression profile. Changes in gene expression patterns on liver abscess formation were not consistent between clinical and laboratory strains.

## Author summary

Various genotypes of *Entamoeba histolytica* are prevalent in the field. Some papers suggest the association between genotypes and disease severity. However, most studies for assessing pathogen virulence were performed using laboratory strains, such as virulent HM1: IMSS (HM1) and avirulent Rahman, because axenic isolation from clinical specimen is technically complex and time consuming. This transcriptomic analysis using clinical strains from the patients with different clinical severity, as well as the laboratory strain HM1 under different conditions showed unique gene expression patterns. Following things were confirmed; 1. Virulent clinical strain maintains its virulence with unique gene expression pattern after axenic isolation, 2. Continuous environmental stress enhances its virulence via the accumulation of altered gene expressions, and 3. Changes in gene expression on the liver abscess formation are not always the same amongst strains. For an accurate understanding the pathogenesis, comprehensive analyses of various clinical strains under different environmental conditions should be promoted.

## Introduction

*Entamoeba histolytica*, the causative agent of invasive amebiasis, is the second most common intestinal parasitic cause of mortality worldwide [1]. The severity of *E*. *histolytica* infection varies. Although most infected individuals display self-limiting diarrhea at an early phase followed by asymptomatic chronic infection, 10% of infected individuals develop “symptomatic” invasive diseases, including life-threatening fulminant amebiasis [2, 3]. Three main factors are known determinants for disease severity of amebiasis, these are host genetic factors, environmental factors, and pathogen virulence factors [4]. There is also interplay between these factors. Whole genome analysis of *E*. *histolytica* was completed in 2005 on the most commonly used laboratory strain, HM-1:IMSS (HM-1) [5]. The virulence genes and their changes in expression have mostly been analyzed using HM-1 strain under various artificial environmental conditions [6, 7], although some studies use another avirulent laboratory strain (Rahman strain) for comparison with HM-1 [8–10]. However, molecular epidemiological studies have shown that various genotypes of *E*. *histolytica* are prevalent even in the same geographical location [11, 12]. Furthermore, some epidemiological studies have suggested the association between specific genotypes of *E*. *histolytica* and disease severity [13, 14]. Moreover, it remains unclear whether observations in laboratory strains are applicable universally, but despite this, studies using clinical strains of *E*. *histolytica* are rare because axenic isolation of *E*. *histolytica* strains from clinical samples is technically complex and time-consuming. To fill this knowledge gap, we recently launched a project for the collection of clinical strains of *E*. *histolytica*, and initiated genomic analysis of these strains [15].

In the present study, *E*. *histolytica* from patients presenting with different severities of amebiasis were isolated as axenically-cultured strains. We assessed their virulence, and the gene expression profiles during *in vitro* passage and liver abscess formation.

## Results

### Isolation of clinical *E*. *histolytica* strains from patients showing different degrees of severity of amebiasis

**Fig 1:**
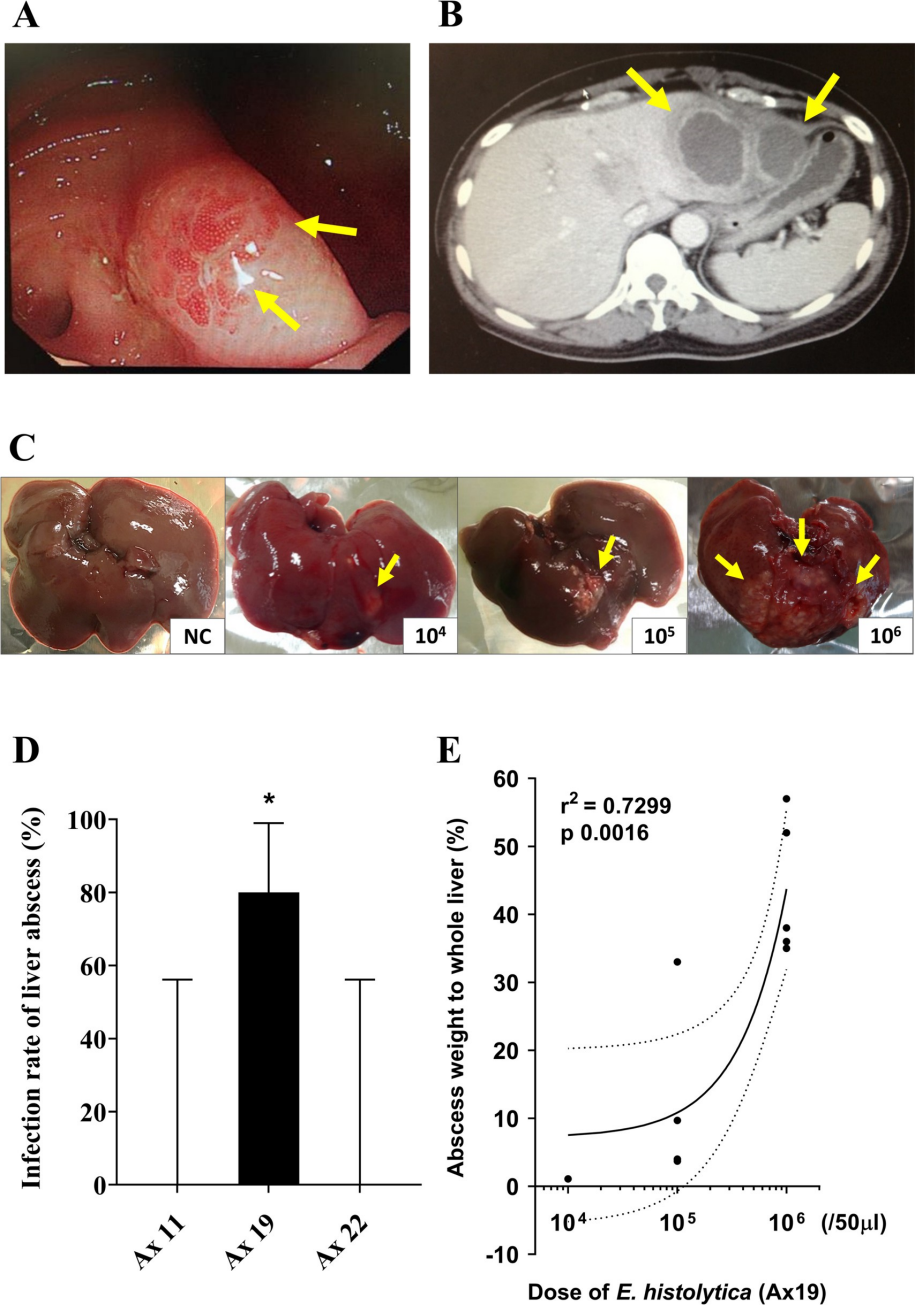
Comparison of the virulence potential of isolated *E*. *histolytica* clinical strains using an animal model. (A) Colonoscopy image of asymptomatic chronic infection. Multiple erosions localized in the cecum were identified (yellow arrows). (B) CT finding of multiple amebic liver abscesses (yellow arrows). *E*. *histolytica* clinical strain Ax19 was isolated from the aspirated pus from the abscess. (C) Experimental amebic liver abscess produced by injection of a highly virulent strain (Ax19) into Syrian hamsters. The size of the liver abscess increased according to the injected dose of Ax19. NC, negative control. (D) Each challenged hamster was euthanized 7 days after *E*. *histolytica* injection. *E*. *histolytica* infection was defined as a positive result following *in vitro* culture of pieces of the resected liver (* p-value < 0.05). (E) Proportion of liver abscess to whole liver in weight for the highly virulent strain (Ax19). The proportion was positively correlated with the dose of Ax19 injected in 50 μl for an average 60 g hamster. Error bars show the standard error of the mean. Statistical significance is indicated.

To assess the impact of pathogen virulence on the clinical severity of *E*. *histolytica* infection, *E*. *histolytica* clinical strains were isolated from the clinical specimens of three patients (clinical strains: Ax11, 22, and 19). First, the asymptomatic strain (Ax11) was isolated from aspirated intestinal fluid collected during colonoscopy. For this asymptomatic patient, *E*. *histolytica* infection was initially suspected because of a positive result in a serum antibody screening test at diagnosis for other sexually transmitted infections. Endoscopy detected a few tiny intestinal erosions located only in the cecum (Fig 1A). *E*. *histolytica* infection was confirmed by polymerase chain reaction (PCR) of the aspirated intestinal fluid. Second, the colitis strain (Ax22) was isolated from the diarrheal stool sample of a HIV-positive male patient who developed vomiting, abdominal pain, and diarrhea, lasting a few weeks. *E*. *histolytica* infection was confirmed by PCR of a stool sample. All clinical symptoms were improved by metronidazole monotherapy. Third, the liver abscess strain (Ax19) was isolated from the aspirated pus from the liver abscess of a female patient (Fig 1B). She developed fever, chills, loss of appetite, abdominal pain, and diarrhea lasting a few days. Serum antibody testing for *E*. *histolytica* antibody was positive. *E*. *histolytica* infection was confirmed by PCR of the aspirated pus from the liver abscess.

**Fig 2:**
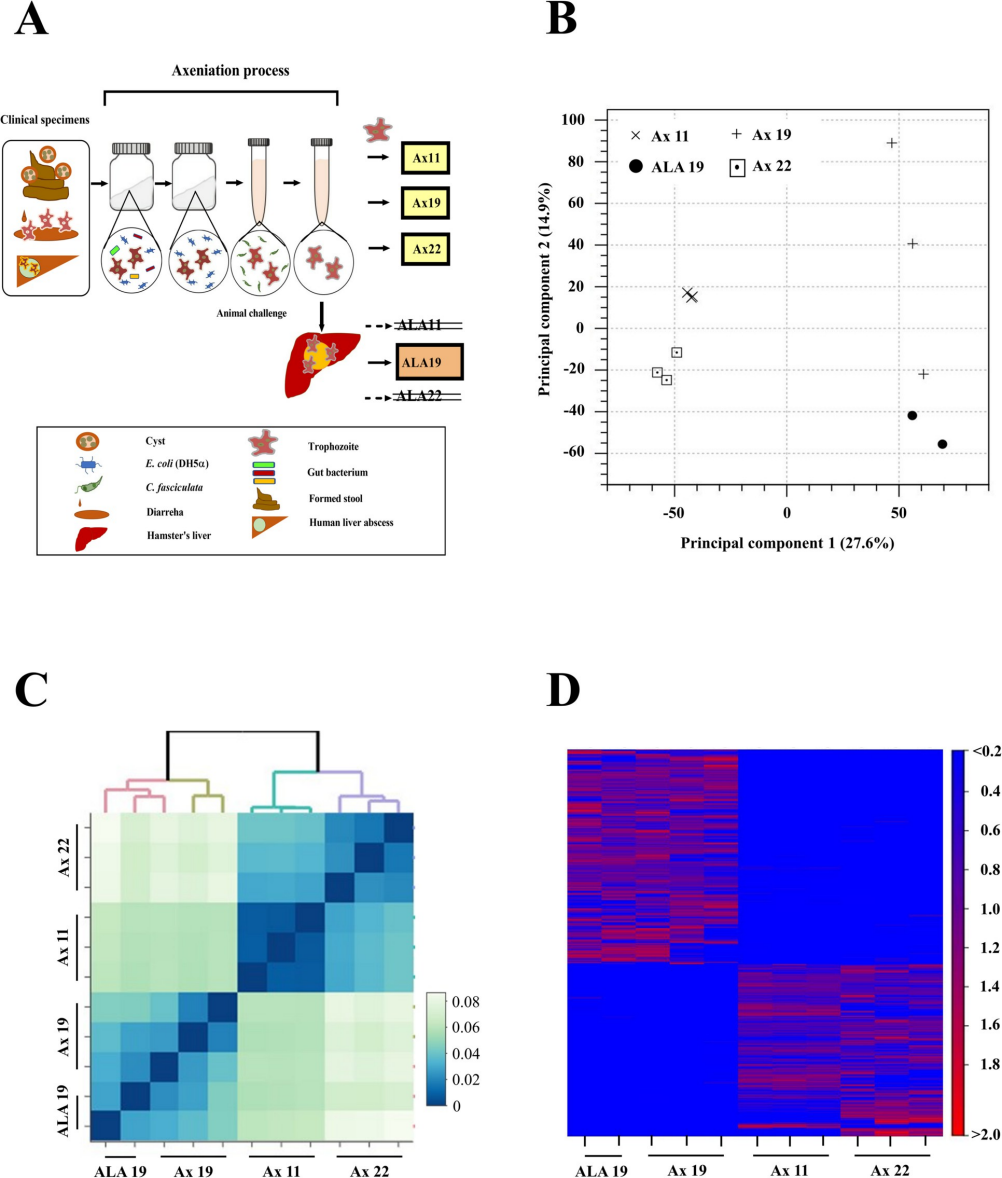
Transcriptome profiling of RNA-seq reads in the isolated clinical strains. (A) Workflow for the RNA-seq experimental procedure. The *E*. *histolytica* clinical strains were isolated from each clinical specimen. First, they were incubated under xenic conditions with *E*. *coli* and rice starch in Robinson’s medium for several weeks to reduce human gut bacteria gradually. After adaptation to the xenic culture, the parasites were next transferred to monoxenic culture medium with *Crithidia fasciculata*. Finally, the parasites were maintained in axenic culture without any bacteria. Animal experiments involved injecting axenically-cultured clinical strains to assess the parasite’s virulence in terms of liver abscess formation. Liver abscesses were successfully formed only when Ax19 (liver abscess strain) was injected. Total RNA was extracted from the trophozoites of the *in vitro*-cultured strains (Ax11, Ax19, and Ax22) and the animal-passaged strain (ALA19). (B) Two-dimensional (2D) plot showing principal components analysis (PCA) of the RNA-seq reads. Each data point represents a read, with the three isolated clinical strains being analyzed in triplicate. The expression pattern of the Ax19 strain is distinct from those of other two strains. (C) Hierarchical clustering based on the Spearman rank-based clustering of the three strains. (D) Heat map showing the clustering of the three strains based on the Pearson’s correlation coefficients using expression values.

After 10, 26, and 25 weeks, respectively, of axenization (Fig 2A), the Ax11, Ax19, and Ax22 *E*. *histolytica* clinical strains were successfully established. For these axenically-cultured clinical strains of *E*. *histolytica*, we performed genotyping of the sequence of six loci of non-coding short tandem repeats (STR) in the intergenic region associated with transfer RNA genes (Table 1). According to the genotype classification in previous reports, strains Ax19 and Ax22 were J9 and J8, respectively [11]. Strain Ax11 showed a unique STR in the D-A locus, while the STR patterns in the other five loci were the same as J9 (S1 Data). Thus, genetically distinct clinical strains were successfully isolated from the patients showing different clinical forms of *E*. *histolytica* infection.

**Table 1:** Genotypes of the three isolated clinical strains as determined using transfer RNA-linked short tandem repeats.

Strain	Sequence type						Genotype^a^
	D-A	A-L	N-K2	R-R	S^TGA^-D	S-Q	
Ax11	***J5DA*** ^b^	4AL	1NK	6RR	15SD	4SQ	***J24*** ^***b***^
Ax19	15DA	J8AL	J3NK	5RR	12SD	J1SQ	J9
Ax22	5DA	4AL	1NK	6RR	15SD	4SQ	J8

^a^ Genotype of each strain refers to a previous report [11].

^b^ A unique sequence type in the D-A locus and a new genotype of Ax11 strain are shown in bold/italics/underlined.

### *In vivo* virulence of clinical strains

To determine the virulence of each clinical strain, we injected the livers of Syrian hamsters with each strain (Ax11, 19, or 22), and assessed liver abscess formation. Murine colitis model is another possible experimental model to assess the pathogen virulence, however, contamination of gut microbiome to the axenic culture media from the murine intestine might influence gene expression of *E*. *histolytica*. Liver abscess model of Syrian hamster was chosen in the present study. First, 10^5^ trophozoites were used for the liver challenge. Liver abscess lesions, which contained live *E*. *histolytica*, were only detected in the hamsters injected with Ax19 (liver abscess strain) (Fig 1C and 1D). A positive correlation was found between the challenge dose of Ax19 and the size of the amebic liver abscess (Fig 1E). Whereas, no liver abscess lesions were detected in hamsters injected with the Ax11 or Ax22 strain, even after challenge with a higher dose of trophozoites. Based on the results of animal experiments, it was indicated that the virulence of the pathogen (Ax19) played an important role in determining clinical severity in this patient, and that its virulence was maintained for several months of axenization.

### Differences in the RNA expression of clinical strains under *in vitro* passage and on liver abscess formation

First, to determine the gene expression profile of each clinical strain under axenic culture conditions, we performed transcriptome analysis of three clinical strains of *E*. *histolytica* during *in vitro* passage (Fig 2A). We collected messenger RNA (mRNA) from trophozoites of the three strains that had been axenically-cultured in YIMDHA-33 culture media to log phase soon after the completion of the axenization. After preparing complementary DNA (cDNA) from the extracted mRNA, RNA-seq was performed. Average clean read numbers of 13.6, 12.3, and 12.2 million were derived from the Ax11, Ax19, and Ax22 strains, respectively. In principal component analysis (PCA), the reproducibility of each strain and differentiation among strains were confirmed using independently collected triplicate RNA-seq data. The reproducibility of the data obtained with strains Ax11 (asymptomatic strain) and Ax22 (colitis strain) was improved compared with that of virulent clinical strain Ax19 (liver abscess strain) (Fig 2B). Hierarchical cluster analysis using Spearman’s correlations showed that the RNA expression pattern of the Ax19 virulent strain was distinct from those of the other two strains (Fig 2C). Next, to determine changes in the RNA expression profile in response to liver abscess formation, we collected RNA from *E*. *histolytica* culture after passage in the liver for strain Ax19 (ALA 19). Interestingly, the difference in the RNA expression profile between Ax19 and ALA 19 was less significant than between Ax19 and other clinical strains (Fig 2 showing the PCA and heatmap). Taken together, each clinical strain was found to maintain a distinct gene expression pattern under the same *in vitro* culture conditions for more than several months of axenization, and the differences observed were greater than the changes induced by environmental stress during liver challenge.

### Differentially expressed genes (DEGs) in a virulent strain (Ax19) during *in vitro* passage

**Fig 3:**
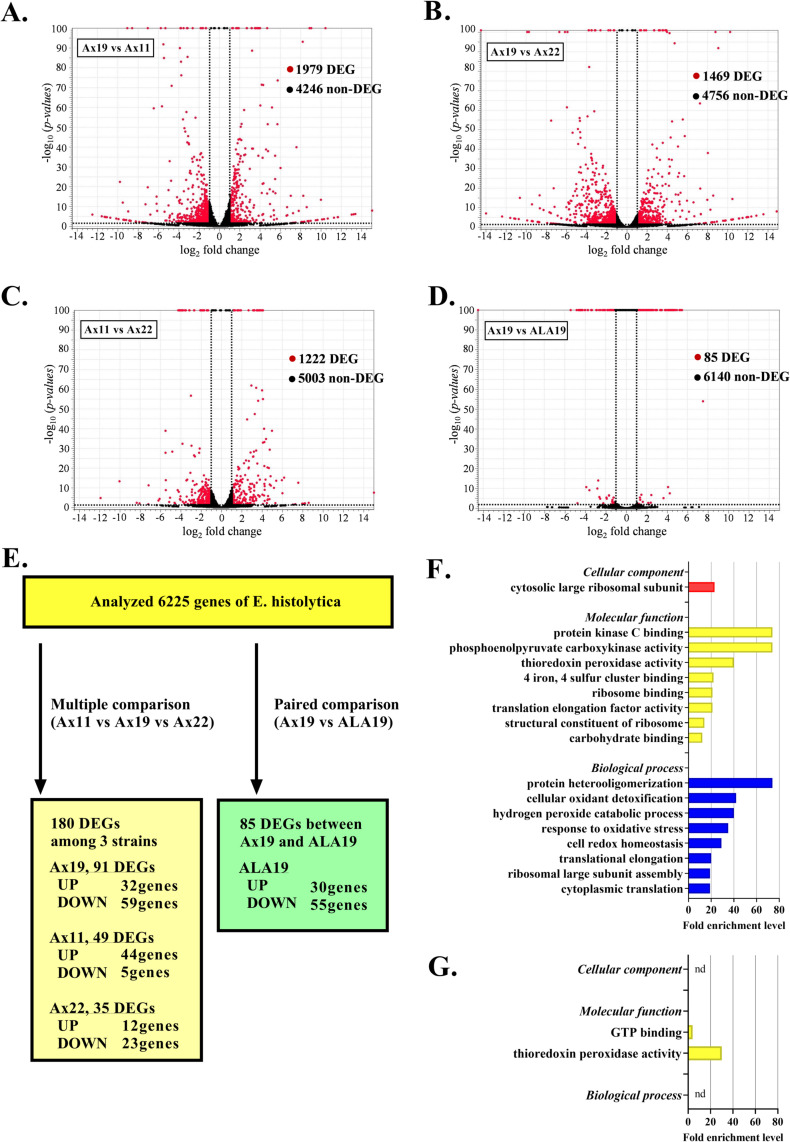
Systematic comparison and assessment of differentially expressed genes (DEGs) between the highly virulent strain (Ax19) and the strains of low virulence (Ax11 and Ax22). (A–D) Volcano plot showing each DEG among the *E*. *histolytica* clinical strains. The vertical axis (y-axis) corresponds to the level of significance of each gene value at log 10 (p-value), and the horizontal axis (x-axis) displays the log 2-fold change value. The red dots represent the DEGs; the black dots represent the non-DEGs. Dotted lines indicate cutoffs; fold changes greater than 2 or less than 0.5; p-value < 0.05. The greatest number of DEGs (1,979 genes) was identified between Ax19 and Ax11. (E) Results of DEG comparisons to select *E*. *histolytica* clinical strain- and environment-specific DEGs. To investigate *E*. *histolytica* clinical strain-specific DEGs further, we selected 180 DEGs from the 6,225 *E*. *histolytica* genes using the Benjamini and Hochberg method with a false discovery rate (FDR) of 5%, followed by Tukey’s multiple comparison test, for three clinical strains. Of the 180 DEGs, each strain-specific DEG was identified as an up-regulated (top) and or down-regulated (bottom) gene using the multiple comparison method. We also selected 85 DEGs by pairwise comparisons between the *in vitro*-cultured Ax19 strain and the liver-passaged ALA19 strain to detect Ax19 environment-specific DEGs. (F) Gene ontology (GO) functional classification. Using PANTHER tools to analyze the biological functions of the 91 Ax19 strain-specific DEGs, we identified 17 GOs in first level categories, including one GO in cellular components (red bar), eight GOs in molecular function (yellow bars), and eight GOs in biological processes (blue bars). (G) Gene ontology (GO) functional analysis using the PANTHER tool for the 85 Ax19 environment-specific DEGs. Unlike the functional analysis of Ax19 strain-specific DEGs, only two GOs in molecular function were detected for the Ax19 environment-specific DEGs, with no statistical enrichments in biological processes and cellular components.

Analyzing the RNA-seq data of *E*. *histolytica* clinical strains revealed a total of 12,375 transcripts. Differentially expressed genes (DEGs) were defined as genes with a < 5% false discovery rate following the statistical analysis performed using the CLC Genomics Workbench (Fig 3A–3D, see details in Materials and Methods). First, we performed pairwise comparisons of different clinical strains and compared the same strain (Ax19) before and after liver challenge using a suite of algorithms (a negative binomial generalized linear model within the CLC Genomics Workbench). The number of DEGs identified by comparison of Ax19 with strain Ax11 was 1,979 (Fig 3A) and with strain Ax22 was 1,469 (Fig 3B), both of which were higher than that from the comparison between Ax11 and Ax22 (Fig 3C, 1,222 DEGs) (S2 Data). Interestingly, only 85 DEGs were identified from the paired comparison before and after liver challenge (Fig 3D, Ax19 vs ALA 19).

Next, to identify specifically expressed/suppressed genes in the virulent strain, we compared the gene expression profile of Ax19 with those of the other two strains. Using two different multiple comparison methods (FDR multiple ANOVA and Tukey’s comparison analysis), 180 DEGs were identified among the three strains. Of these 180 genes, Ax19 strain-specific DEGs were defined as genes whose expression was significantly up- or down-regulated in Ax19 compared with the other two strains. Finally, we identified 91 Ax19 strain-specific DEGs, including 32 up- and 59 down-regulated genes (S3 Data). In addition, 49 DEGs (44 up-regulated and 5 down-regulated) and 35 DEGs (12 up-regulated and 23 down-regulated) were identified as Ax11 and Ax22 strain-specific DEGs, respectively (S3 Data). Interestingly, 26 strain-specific DEGs were common between strains Ax19 and Ax11, but were inversely up- or down-regulated between the two strains (Table 2).

**Table 2:** Orthologous lists of the 26 differentially expressed genes that were oppositely up- or down-regulated between Ax19 (liver abscess strain) and Ax11 (asymptomatic strain).

Gene ID name	Function prediction			Fold changes
	Orthology	PANTHER family	PANTHER protein class	Ax19/Ax11
EHI_001420	Thioredoxin domain-containing protein	Peroxiredoxin-4	Peroxidase (PC00180)	0.178
EHI_006980	Gal/GalNAc lectin Igl1	TNFR-Cys domain-containing protein	ND	0.575
EHI_010650	Ribosomal_L30 domain-containing protein	60S ribosomal protein L7-related	Ribosomal protein (PC00202)	0.621
EHI_017700	60S ribosomal protein L13, putative	60S ribosomal protein L13A	Ribosomal protein (PC00202)	0.513
EHI_029620	Aldose reductase, putative	Aldo-keto reductase family 1 member A1	Reductase (PC00198)	0.368
EHI_030750	PPi-type phosphoenolpyruvate carboxykinase 2	ND	ND	0.382
EHI_042370	Galactose-specific adhesin 170 kD subunit, putative	ND	ND	0.263
EHI_044810	Ribosomal_L16 domain-containing protein	60S ribosomal protein L10	Ribosomal protein (PC00202)	0.461
EHI_050550	WD_REPEATS_REGION domain-containing protein	Receptor for activated C kinase 1	ND	0.670
EHI_068200	60S ribosomal protein L31, putative	60S ribosomal protein L31	Ribosomal protein (PC00202)	0.579
EHI_116360	Serine-rich protein	RIKEN cDNA 4932415D10 gene	ND	0.689
EHI_122310	Thioredoxin domain-containing protein	Peroxiredoxin-4	Peroxidase (PC00180)	0.075
EHI_133900	Galactose-inhibitable lectin 170 kDa subunit, putative	ND	ND	0.474
EHI_140120	Actin	Actin	Actin and actin-related protein (PC00039)	0.475
EHI_146110	Uncharacterized protein	ND	ND	0.623
EHI_150470	Ribosomal_L2_C domain-containing protein	60S ribosomal protein L8	Ribosomal protein (PC00202)	0.519
EHI_159160	Superoxide dismutase	Sod_Fe_C domain-containing protein	Oxidoreductase (PC00176)	0.265
EHI_159480	Pore-forming peptide ameobapore A, putative	ND	ND	0.598
EHI_160930	PALP domain-containing protein;cysteine synthase type II	Cysteine synthase 1	Lyase (PC00144)	0.314
EHI_160980	Uncharacterized protein	ND	ND	0.713
EHI_177630	60S acidic ribosomal protein P0	60S acidic ribosomal protein P0	Ribosomal protein (PC00202)	0.718
EHI_182900	Actin	Actin	Actin and actin-related protein (PC00039)	0.278
EHI_182920	60S ribosomal protein L21, putative	60S ribosomal protein L21	Ribosomal protein (PC00202)	0.421
EHI_201250	Thioredoxin domain-containing protein	Peroxiredoxin-4	Peroxidase (PC00180)	0.236
Tr*	ND	ND	ND	1.426
EhSINE1_25*	ND	ND	ND	0.632

Abbreviations: DEG, differentially expressed gene; ND, no data; PC, protein class.

**Fig 4:**
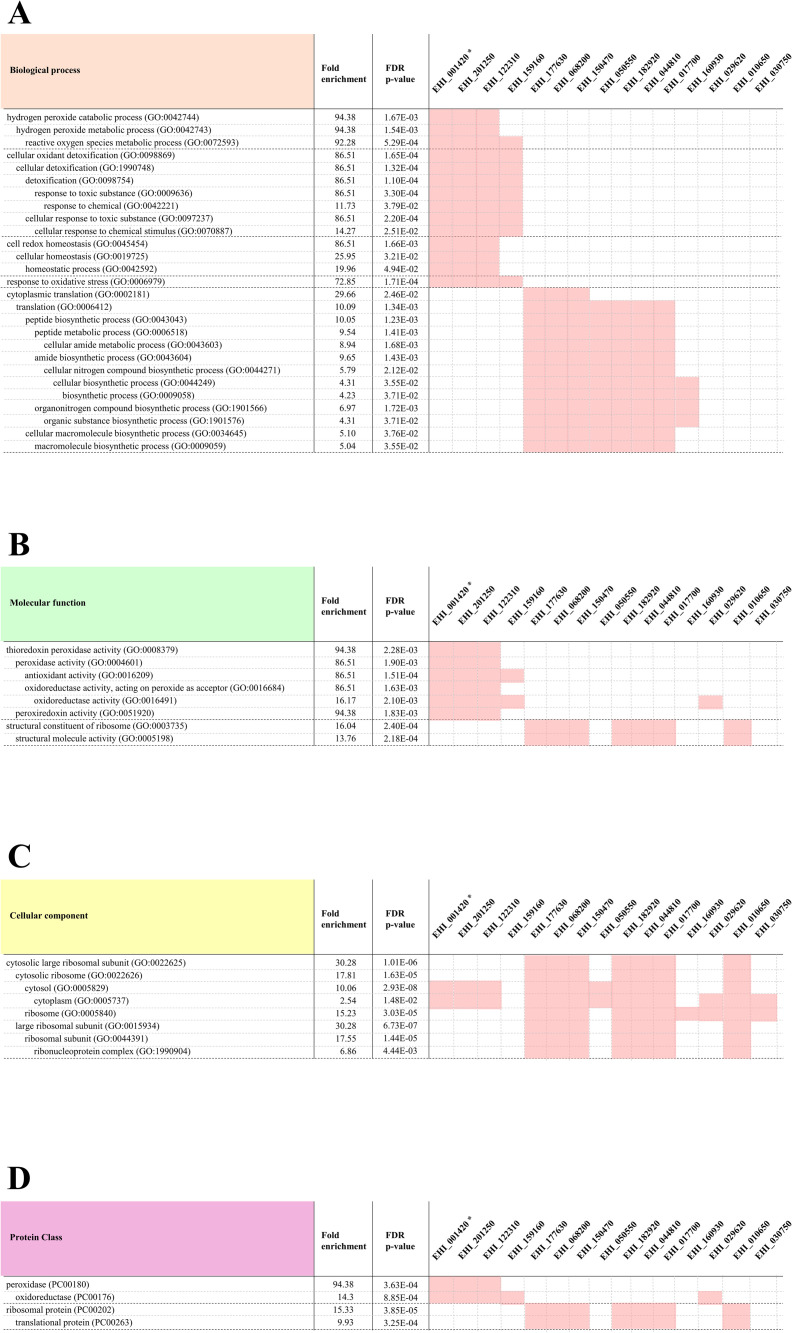
Clustering of 15 multi-functional genes among the 26 DEGs that are inversely up- or down-regulated between strains Ax19 and Ax11. (A) Biological process. (B) Molecular function. (C) Cellular component. (D) Protein Class. *EHI_001420 gene was identified in the gene lists for five overlapping genes between the 91 Ax19 strain-specific DEGs and the 85 Ax19 environment-specific DEGs (S9 Data).

Next, to investigate the impact of strain-specific DEGs on the biological function of *E*. *histolytica*, we applied the PANTHER classification system. Enrichment analysis was performed to identify gene ontology (GO) categories and protein classes (PCs) that were significantly influenced by the DEGs identified in this study. GOs were sorted into the different subcategories for biological processes (BP), molecular function (MF), and cellular component (CC). Enrichment was defined as the ratio of frequency of GO-related genes in DEGs compared with that expected from the PANTHER database. High enrichment indicates that functional genes are more frequently detected among the DEGs of interest than the number expected from the reference data based on the Ensemble gene list, including HM1, Rahman strain, and some clinical strains. Among the 91 strain-specific DEGs identified for the Ax19 virulent strain, 80 genes (87.9%) were mapped as functional genes of *E*. *histolytica* in the PANTHER database (S4 Data). We identified 17 GOs (one CC, eight MFs, and eight BPs) and two PCs as highly enriched in biological function (Fig 3F and S5 Data). Of the Ax11 strain-specific DEGs, 91.8% (45/49) were mapped in the PANTHER database (S4 Data). We identified eight GOs (one CC, two MFs, and five BPs) and two PCs as enriched in biological function (S6 Data). Although 94.3% (33/35) of the Ax22 strain-specific DEGs were mapped in the PANTHER database, no enrichment of biological functions was identified from these DEGs (S7 Data). Interestingly, all of the Ax11-related enriched biological functions (eight GO categories and two PCs) were shared by strain Ax19. Moreover, it was confirmed that enrichment analysis using 26 DEGs, which are inversely up- or down-regulated between Ax19 and Ax11, completely matched the results obtained using Ax11 strain-specific DEGs (Table 2). In particular, 15 genes, with multiple functions, had strongly represented among the results of PANTHER enrichment analysis (Fig 4 and S8 Data). Taken together, these findings confirm that distinctive gene expression profiles in the clinical strains during *in vitro* passage are associated with their biological activities. Our findings also strongly indicate that two distinct clinical strains isolated from patients with opposing clinical severity (Ax11: asymptomatic strain, and Ax19: liver abscess strain) showed opposing biological behavior during *in vitro* passage.

### Changes in gene expression on liver challenge

Next, to investigate alterations in gene expression in response to environmental changes induced by liver challenge, we analyzed 85 DEGs, which were identified from a paired comparison before and after liver challenge, as Ax19 environment-specific DEGs (Fig 3E). Only five genes were shared between the strain-specific (91 genes) and environment-specific (85 genes) DEGs of Ax19. Furthermore, two up-regulated and three down-regulated strain-specific DEGs were recognized as inversely down- and up-regulated by liver challenge. Thus, none of the strain-specific DEGs were regulated in the same way following liver challenge.

Among the 85 environment-specific DEGs of strain Ax19, 71 genes (83.5%) were mapped in the PANTHER database. However, only two GOs (MF), and two PCs were identified as enriched in biological functions. Among them, one GO (thioredoxin peroxidase activity) and one PC (peroxidase) overlapped with those of Ax19 strain-specific DEGs. These results indicate that environment-specific DEGs following liver challenge have less impact on biological function than strain-specific DEGs of Ax19, although peroxidase activity has previously been reported as a representative biological function related to virulence that is affected [16].

### RNA expression of laboratory strain HM-1:IMSS clone 6 under different conditions

**Fig 5:**
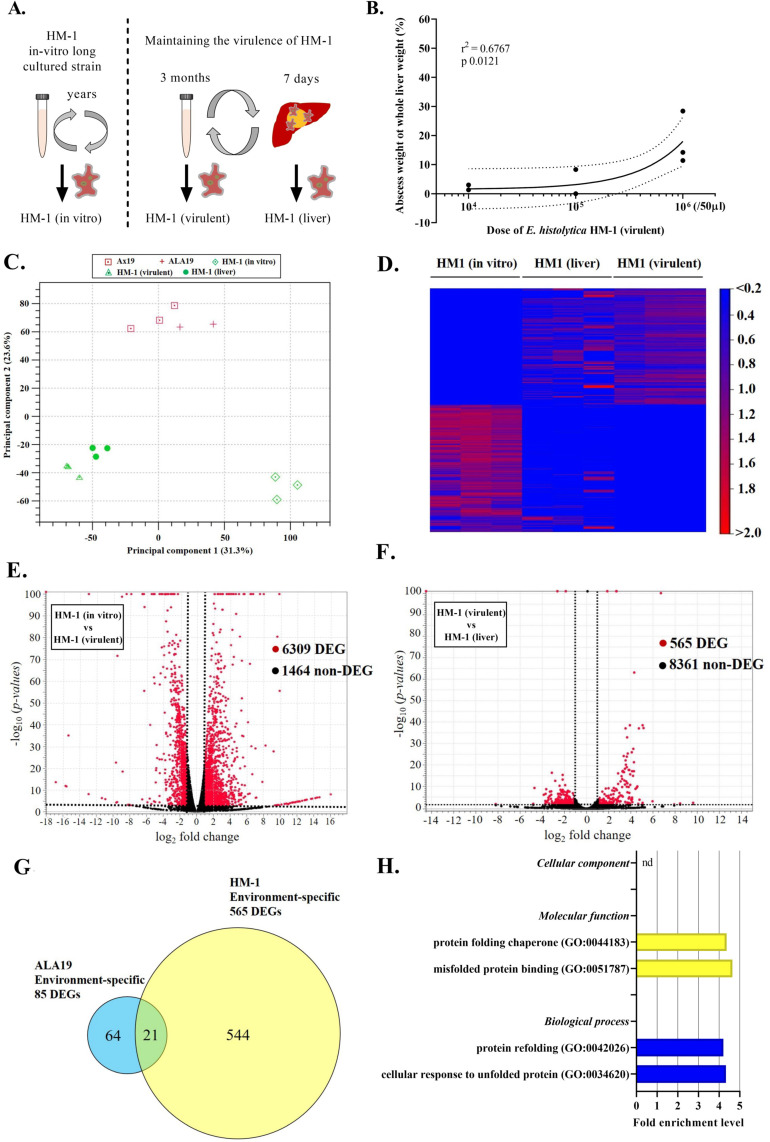
Transcriptome profiling of the *E*. *histolytica* laboratory strain (HM-1:IMSS clone 6). (A) Experimental work flow for the three different culture conditions: HM-1 (*in vitro*), maintained under *in vitro* culture conditions for many years; HM-1 (virulent), maintained under virulent conditions by routinely passaging through an animal liver every 3 months; HM-1 (liver), collected just after liver challenge with strain HM-1. (B) The proportion of liver abscess weight to whole liver weight for strain HM-1 (virulent). The proportion was positively correlated with the dose of HM-1 (virulent), but the linear relationship of HM-1 (virulent) was relatively weak compared with that of the highly virulent Ax19 strain. (C) 2D plot of principle component analysis (PCA) of RNA-seq reads from the three different culture conditions for strain HM-1, using the two different culture conditions for the highly virulent Ax19 strain as reference gene profiles. Each data point represents a read, analyzed in triplicate. The expression profile of HM-1 (*in vitro*) was clearly different from that of HM-1 (virulent). The difference was greater than the changes induced by liver challenge (HM-1 (virulent) vs HM-1 (liver)). Moreover, the expression profile of HM-1 (virulent) was also distinct from that of ALA19. (D) Heat map of the three different culture conditions for the HM-1 strain. The expression profile of HM-1 (*in vitro*) was clearly different from those of HM-1 (virulent) and HM-1 (liver). (E and F) Volcano plots showing each differentially expressed gene (DEG) for the *E*. *histolytica* laboratory strain. Although there were 6,309 DEGs identified between HM-1 (*in vitro*) and HM-1 (virulent), there were no more than 565 DEGs identified between HM-1 (virulent) and HM-1 (liver). (G) Venn diagram of the 85 Ax19 environment-specific DEGs and the 565 HM-1 environment-specific DEGs. The number of overlapping genes was 21. (H) GO function analysis of the 565 HM-1 environment-specific DEGs.

Our results have shown that each clinical strain isolated from cases of different clinical severity presents a distinct gene expression profile even during *in vitro* passage. On the other hand, in the case of *E*. *histolytica* laboratory strains, we commonly perform animal challenge every 2 to 3 months to ensure virulence is maintained. From this, we infer that intermittent environmental stress can alter the gene expression pattern relating to virulence, and this change can last for several months. Therefore, to observe the long-term effect of intermittent environmental stress on the *in vitro* gene expression profile of *E*. *histolytica*, we performed transcriptome analysis of the single laboratory *E*. *histolytica* HM-1 strain (clone 6) [17] under different conditions, and compared the DEGs. We prepared cDNA for RNA-seq from HM-1:IMSS clone 6 under the following three conditions (Fig 5A): [1] HM-1 (*in vitro*): HM-1:IMSS was maintained in *in vitro* culture media, [2] HM-1 (virulent): HM-1:IMSS was maintained in the same media, but passaged in hamster liver every 3 months, and [3] HM-1 (liver): HM-1:IMSS was collected just after liver challenge with HM-1 (virulent). In our laboratory, HM-1 (virulent) is cultured with *Crithidia fasciculata* (monoxenic culture) to maintain its virulence. We confirmed that in vitro gene expressions were influenced by co-culturing with *C*. *fasciculata* (S1 Fig). Therefore, all three HM-1 (HM-1 (in vitro), HM-1 (virulent), and (HM-a (liver)) were maintained under the same conditions with *C*. *fasciculata*, and their gene expression profiles were compared.

First, we confirmed that HM-1 (virulent) induces the formation of liver abscesses in a dose-dependent manner (Fig 5B). However, the size of the liver abscess induced by HM-1 (virulent) was significantly smaller compared with that of Ax19, when using 10E6 trophozoites for the challenge. In PCA, the gene expression profile of HM-1 (*in vitro*) was clearly distinct from that of HM-1 (virulent), and the degree of difference was the same as between HM-1 (*in vitro*) and Ax19 virulent strain, although the culture conditions differed between HM-1 (monoxenic culture) and Ax19 (axenic culture) (Fig 5C, Ax19 and ALA 19 were plotted as reference data). Moreover, the difference between HM-1 (*in vitro*) and HM-1 (virulent) was even greater than the change induced by liver challenge (HM-1 (virulent) vs HM-1 (liver)), which was consistent with the results from clinical strain (Ax19). Next, DEGs were calculated by two different types of pairwise comparisons: [1] DEGs between HM-1 (*in vitro*) and HM-1 (virulent) as HM-1 strain-specific DEGs, and [2] DEGs between HM-1 (virulent) and HM-1 (liver) as HM-1 environment-specific DEGs (Fig 5E and 5F). Surprisingly, 81.2% of the analyzed genes (6,309 out of 7,774 genes) were differentially expressed between HM-1 (*in vitro*) and HM-1 (virulent). By contrast, RNA expression was altered by liver challenge in only 6.3% of cases (565 out of 8,917 genes). Taken together, periodic, repeated liver challenge of the laboratory strain altered and maintained not only its virulence but also the gene expression profile. Furthermore, these changes accumulate as a result of repeated environmental stress.

Finally, to assess the applicability of the findings from animal experiments with a single laboratory strain to other strains, we compared environment-specific DEGs between HM-1 and Ax19. As shown in Fig 5G, 85 genes and 565 genes were identified as Ax19- and HM-1-environment-specific DEGs, respectively, of which, only 21 genes overlapped. Of these 21 DEGs, only nine were up- or down-regulated in the same manner. As shown in Table 3, eight of these nine genes were annotated in the *E*. *histolytica* database. Twelve genes were inversely up- or down-regulated between the two strains. Furthermore, PANTHER enrichment analysis using 565 HM-1 environment-specific DEGs, identified two GOs (MF), and showed completely different results from those obtained using Ax19 environment-specific DEGs (Fig 5H). Taken together, gene expression changes induced by liver challenge in a hamster model are highly dependent on the type of strain, and liver abscess formation can be linked with different gene expression profiles.

**Table 3:** Orthologous lists of the nine genes that were similarly up- or down-regulated among the environment-specific DEGs of strains Ax19 and HM-1 (virulent).

Gene ID name	Function prediction			Environment-specific DEGs			
	Orthology	PANTHER family	PANTHER protein class	Ax19 strain		vHM-1 strain	
				Fold change	FDR p value	Fold change	FDR p value
EHI_067950	Protein-tyrosine-phosphatase	CDC25-like protein phosphatase twine related	Protein phosphatase (PC00195)	0.226	0.043	0.387	9.8E-03
EHI_014280	Uncharacterized protein	VWFA domain-containing protein-related	ND	0.531	0.008	0.466	0.031
EHI_004340	Serine-threonine-isoleucine rich protein, putative	ND	ND	0.511	4.7E-04	0.288	1.3E-08
EHI_109690	Glycerophosphocholine acyltransferase 1	Glycerophosphocholine acyltransferase 1	ND	3.58	1.5E-07	1.49	0.020
EHI_184500	Uncharacterized protein		Guanyl-nucleotide exchange factor (PC00113)	23.8	3.4E-08	3.00	0.014
EHI_114950	Uncharacterized protein	AIG1-type domain-containing protein-related	Cytoskeletal protein (PC00085)	0.332	4.0E-06	0.128	3.6E-13
EHI_077750	Uncharacterized protein	ND	ND	0.080	2.5E-11	0.095	3.0E-08
EHI_073060	Uncharacterized protein	AIG1-type domain-containing protein-related	Cytoskeletal protein (PC00085)	0.101	8.7E-10	0.261	1.5E-06
EHI_C00051	ND	ND	ND	5.60	2.6E-03	3.63	6.6E-03

Abbreviations: DEG, differentially expressed gene; ND, no data; PC, protein class; vHM-1, HM-1 (virulent).

## Discussion

Most previous studies on the virulence of *E*. *histolytica* have used laboratory strains, such as virulent HM-1:IMSS and avirulent Rahman, and have assessed changes in gene expression under artificial environmental stresses [18, 19]. This study is the first to compare the gene expression profiles of live *E*. *histolytica* strains isolated from patients with disease of different clinical severity. Originally, we planned to compare the changes in gene expression before and after the liver abscess challenge of the hamsters (environmental-specific DEGs) among different clinical strains. However, only strain Ax19 derived from liver abscess patient induced liver abscesses in the hamster model. Therefore, we first compared the gene expression of different strains *in vitro*. Surprisingly, the gene expression profile of Ax19 was clearly distinct from the other two strains, as represented by the Ax19 strain-specific DEGs. Also, PANTHER databases suggested that many biological functions (17 GOs and 2 PCs) are differentially expressed in the Ax19 virulent strain. Importantly, 26 of the Ax19 strain-specific DEGs were oppositely up- or down-regulated in the Ax11 avirulent strain, in which we identified 15 determinant genes with overlapping functions (8 GOs and 2 PCs overlapped between Ax11 and Ax19). Taken together, the virulent or avirulent phenotype of the clinical strain is well-characterized by the gene expression profile on *in vitro* passage, despite several months of axenization. We also detected changes in gene expression before and after liver challenge (environment-specific DEGs) for strain Ax19. Surprisingly, differences before and after liver challenge were less significant than between any two of the *in vitro* passaged clinical strains. Biological functions related to the Ax19 environment-specific DEGs (2 GOs and 2 PCs) were also fewer than for the Ax19 strain-specific DEGs. In addition, environmental DEGs and the related biological functions of clinical strain (Ax19) rarely overlapped with those of the laboratory strain (HM-1).

Enrichment analysis of the 26 genes that overlapped between Ax19 strain-specific DEGs and Ax11 strain-specific DEGs suggested potential virulent factors affecting clinical severity. As expected, several known virulent functions were detected, including oxidative stress-relating enzymes (EHI_001420, EHI_201250, EHI_122310, EHI_159160), nitrogen compound biosynthetic processing proteins (EHI_177630, EHI_068200, EHI_150470, EHI_050550, EHI_182920, EHI_044810, EHI_017700, EHI_160930), N-acetyl-D-galactosamine inhibitable (Gal/GalNAc) lectin subunit Igl1 (EHI_006980), serine-rich *E*. *histolytica* protein (SREHP) (EHI_116360), and the pore-forming peptide ameobapore A precursor (EHI_159480). To survive and protect against the host immune response, especially nitric oxide and reactive oxygen intermediates, recent reports have suggested that *E*. *histolytica* has effective functional controls in producing peroxiredoxin and thioredoxin systems [6, 20–22]. Moreover, the non-virulent *E*. *histolytica* laboratory strain (Rahman) has been reported to show transcriptional differences and notable biological characters that correlate with sensitivity to H_2_O_2_ stress conditions [8, 9]. Other virulent factors may also play important roles in determining pathogenesis, including factors that code for translational-related, cytoskeletal functions, and dominant surface antigens for adherence to and killing of host cells [23–25]. Taken together, the identified virulent genes in this study using clinical strains were not the same as reported previously using laboratory strains; however, the encoded proteins and their functions overlap considerably between clinical and laboratory strains. In future research, the impact of these genes on the disease severity will be assessed by genetic manipulation models, such as RNA interference and CRISPR/Cas9 [26, 27]. It will also be interesting to study the regulatory pathways and the responses controlled by these DEGs to a variety of stress conditions and stage conversions using various types of clinical strains.

In the present study, the virulence of each strain in a hamster liver abscess model reflects the clinical severity of that strain in the patient from which it was isolated. Also, we previously reported that whole genome analysis of clinical strains revealed significant genomic differences in critical functional genes, such as the AIG1 family genes [28]. Taken together, congenital factors of *E*. *histolytica* play an important role in determining its virulence. However, it remains unclear whether gene expression, which determines virulence, is affected only by congenital genomic factors, or whether it is also influenced by environmental factors. Therefore, we compared gene expression between the *in vitro* maintained laboratory strain HM-1:IMSS clone 6 (HM-1 (*in vitro*)) and the same laboratory strain that has been periodically passaged through a hamster liver (HM-1 (virulent)). The gene expression profile differs between HM-1 (*in vitro*) and HM-1 (virulent), with more than half of the genes presenting as HM-1 strain-specific DEGs. The gene expression patterns during *in vitro* passage were found to be highly altered by periodic liver passage. Interestingly, the number of HM-1 strain-specific DEGs was much higher than the number of gene changes induced by a single liver passage, presented as HM-1 environment-specific DEGs. These results indicate that the virulent phenotype of the laboratory strain can be induced and amplified by periodic animal passage. It also appeared that these characteristics were maintained for at least several months following liver passage, even with subsequent *in vitro* passage. Interestingly, there were very limited commonalities in environment-specific DEGs between the Ax19 clinical strain and the HM-1 laboratory strain, indicating that the gene expression profile of virulence differs between clinical and laboratory strains. In fact, the HM-1 strain has been “in vitro” passaged for a long time after isolation from the clinical specimen. Moreover, it was originally isolated from a diarrheal stool and not from the aspirated liver pas in the colitis patient (colitis strain), which was adapted to the hamster’s liver in the laboratory. Finally, the HM-1 strain has been maintained as a virulent strain under *in vitro* medium with *C*. *fasciculata*. These results emphasize the new biological importance of our analyses using Ax19, which was directly isolated from the patient’s liver abscess. Additionally, differentially expressed genes have been identified by comparing clinically and biologically different *E*. *histolytica* [29]. Thus, it was strongly suggested that continuous environmental stress in addition to predisposed genetic characteristics contribute to the virulence phenotype via alteration of the gene expression profile.

The present study has some limitations. First, we identified 91 strain-specific DEGs from strain Ax19 in the present study. However, this number was significantly lower than that of the HM-1 strain-specific DEGs (6,309 genes). This might be because the Ax19 strain-specific DEGs were calculated after multiple comparisons of the three clinical strains (Ax19, Ax11, and Ax22), whereas those of HM-1 were calculated by a pairwise comparison (HM-1 (*in vitro*) vs HM-1 (virulent)). In addition, the gene expression profile of the *in vitro* passaged laboratory “cloned” strain was more stably reproducible than that of the clinical “crude” strains (Figs 2B and 5C), reflecting the fact that statistical significance was more easily determined in the laboratory strain. However, it is also possible that clinical *E*. *histolytica* strains lose their virulence properties during the several months of axenization. Future studies to analyze a greater number of virulent and non-virulent clinical strains are needed, and alterations in gene expression profiles during axenization should also be analyzed to identify the key virulence genes of *E*. *histolytica*. Second, the reason for the major difference in *in vitro* gene expression between two genetically identical strains (HM-1 (virulent) and HM-1 (*in vitro*)) remains unclear in the present study. One possibility is that drastic genomic changes, which cause an alteration in the expression of more than half of the genes, occurred during periodical liver passage. Whole genome analyses are required to confirm gene homology between the HM-1 (virulent) and HM-1 (*in vitro*) strains, although these strains are derived from the same clone (HM1:IMSS clone 6 strain). Another possibility is that epigenetic modifications may be responsible. DNA methylation and de-methylation of promotor regions can alter the expression of target genes, and some studies have reported that DNA methylation can occur in response to environmental changes, such as oxidative or nitrosative stresses, in *E*. *histolytica* [30, 31]. It may be worthwhile to assess the longitudinal changes in DNA methylation after *in vitro* passage, in addition to changes in virulence and RNA expression.

In conclusion, unique gene expression patterns relating to virulence were well-maintained even after long-term axenization. Virulence gene expression profiles were also influenced by continuous environmental stress. Changes in gene expression that accompany liver abscess formation in virulent strains are not consistent amongst strains. Comprehensive analyses of a wide array of *E*. *histolytica* strains under different environmental conditions are needed to further understand the pathogenesis of *E*. *histolytica* infection.

## Materials and methods

### Ethics statement

This study was approved by the ethics committee of the National Center for Global Health and Medicine (approval no. NCGM-G-001566-02) and was implemented in accordance with the provisions of the Declaration of Helsinki. All animal care procedures were approved by the ethics committee of the National Institutes of Infectious Diseases (approval no. 117155-IV) in accordance with Standards Relating to the Care and Management of Laboratory Animals and Relief of Pain formulated by the Ministry of the Environment.

### Isolation of strains from clinical samples and patient data

*E*. *histolytica* clinical strains were isolated from clinical samples including stool, aspirated intestinal fluid, and aspirated liver abscess samples. These clinical samples were directly collected from patients who were diagnosed with *E*. *histolytica* infection by PCR. After collecting clinical samples, we immediately initiated the isolation steps of xenic culture using the specific cultivation media for *E*. *histolytica*, as previously reported [11, 32]. Briefly, the clinical samples revealing trophozoite forms were directly cultured in Robinson’s R (defined medium for *Escherichia coli*) and BR (R medium precultured with *E*. *coli*) media [33]. In the case of stool samples revealing cyst forms, the samples were treated with 0.1 N HCl for 10 minutes, then washed with fresh water to kill other bacteria and fungi that may affect the cultivation of *E*. *histolytica* before the xenic culture step. Finally, the axenic strains were established from a monoxenic culture with viable *Crithidia fasciculata* (ATCC No. 50083) by the classical approach using YIMDHA-S medium [34, 35]. Clinical data including symptoms and laboratory results were collected at our hospital.

### Experimental amoebic liver abscesses in hamsters

*In vitro*-cultured axenic clinical strains were collected at log phase (60%–80% confluence), and high viability (>90%) was confirmed by trypan blue staining. *E*. *histolytica* cells were counted and resuspended in 100 μl of BI-S-33 medium. Four-week-old male Syrian hamsters were purchased from Japan SLC, Inc. [36]. In total, 10,000–1,000,000 trophozoites of *E*. *histolytica* clinical strains were injected into the left lobe of the liver of Syrian hamsters. The injected animals were euthanized 1 week after injection, and the livers and abscesses were dissected and weighed separately. The concentrated liver pus was added to YIMDHA-S medium. Successful animal infection and liver abscess formation was defined as *in vitro* growth of *E*. *histolytica* in the medium a few days after injection. The independent animal experiments were performed in triplicate.

### E. histolytica reference strains and cultivation

HM-1 (*in vitro*) is an *E*. *histolytica* laboratory strain isolated from HM1:IMSS clone 6 that has been maintained *in vitro* for >10 years [37]. HM-1 (virulent) is the same laboratory strain, which is regularly passaged through liver abscesses of golden hamsters every 3 months. Both strains were cultured monoxenically in YIMDHA-S medium with *Crithidia fasciculata* [34, 35]. Trophozoites of HM1 cultured monoxenically for 4 days after liver abscess formation were analyzed (HM-1 (liver)). Trophozoites of HM1 were monoxenically subcultured under the same conditions with *C*. *fasciculata* for several months (HM-1 (*in vitro*)).

### Diagnostic real-time PCR and genotyping test

To detect *E*. *histolytica* in clinical specimens, a conventional PCR test was performed. Total DNA from clinical specimens was extracted using the QIAamp Fast DNA Stool Mini Kit (Qiagen, Hilden, Germany), whereas the DNAs from amoebic liver abscess patients were extracted directly from abscess samples using a QIAamp DNA Mini Kit according to the manufacturer’s recommended procedures [11]. These DNAs were amplified using primers Ehd-88R and EM-RT-F2, with a 42-nucleotide probe that hybridizes to *E*. *histolytica* amplicons, using the TaqMan Fast Advanced Master Mix 2× buffer (Thermo Fisher Scientific, Waltham, MA, USA) as previously described (95°C for 3 minutes, then 40 cycles of 95°C for 10 seconds and 61°C for 20 seconds) [38, 39]. To identify the *Entamoeba* species in the PCR-positive amplicons, the purified amplicons were sequenced by Sanger sequencing (Eurofins Genomics, Tokyo, Japan). The STR fragments were amplified using six pairs of *E*. *histolytica*-specific tRNA-linked STR primers (DA-H, AL-H, NK2-H, RR-H, SQ-H, and S^TGA^D-H) under the conditions previously described [40]. The amplified PCR products were separated using 1.5% agarose gel (Takara Bio, Tokyo, Japan) and purified using a NucleoSpin Gel and PCR Clean-up kit (Takara). Sequence analysis was performed using appropriate primers by Sanger sequencing (Eurofins Genomics, Tokyo, Japan). Nucleotide sequences were analyzed using ATGC ver. 7 (Genetyx, Tokyo, Japan).

### RNA extraction and sequencing

Total RNA was extracted from approximately 1 × 10^6^ *E*. *histolytica* trophozoites (with each culture performed in triplicate) using a Nucleospin RNA Kit (Takara) according to the manufacturer’s guidance. In short, *E*. *histolytica* trophozoites were collected by centrifugation and then disrupted by the addition of lysis buffer. Genomic DNA was digested by treating with RNase-free rDNase. Total RNA was eluted in a total volume of 50 μl nuclease-free water. The RNA concentration was determined by a Qubit 2.0 Fluorometer using a Qubit RNA BR Assay Kit (Thermo Fisher Scientific). The RNA quality was determined with an Agilent 2100 Bioanalyzer. For *E*. *histolytica* clinical strains, HM-1 (virulent) and HM-1 (liver), library preparation was performed by Eurofins Genomics. The polyA fraction (mRNA) was isolated from total RNA, followed by its fragmentation. Then, double-stranded (ds) cDNA was reverse transcribed from the fragmented mRNA. The ds cDNA fragments were processed for adaptor ligation, size selection (for 200-bp inserts) and amplification to generate cDNA libraries. Prepared libraries were subjected to paired-end 2 × 101 bp sequencing on the HiSeq 2500 and 4000 platform, using the HiSeq 3000/4000 SBS kit. For HM-1 (*in vitro*) strain, library preparation was performed by AZENTA Life Sciences (Tokyo, Japan). The poly(A) mRNA isolation was performed using Oligo(dT) beads. The mRNA fragmentation was performed using divalent cations and a high temperature. Priming was performed using random primers. First-strand and second-strand cDNA were synthesized. The purified ds cDNA was then treated to repair both ends and add a dA-tail in one reaction, followed by a T-A ligation to add adaptors to both ends. Size selection of adaptor-ligated DNA was then performed using DNA Clean Beads. Each sample was then amplified by PCR using P5 and P7 primers and the PCR products were validated. Then, libraries with different indexes were multiplexed and loaded onto an Illumina HiSeq X for sequencing using a 2 × 150 paired-end configuration according to the manufacturer’s instructions.

### Bioinformatic analysis of the RNA-seq data

The RNA-seq reads were trimmed and mapped using the CLC Genomic Workbench (Qiagen) to the *E*. *histolytica* genome assembly (AmoebaDB v1.7, http://amoebadb.org/amoeba/) with a gene model provided by Dr. Hon [41]. The samples with a high transcript integrity number (TIN) over 80 were selected for the following analysis [42]. Orthologs among isolates were identified using the AmoebaDB. Raw fragment counts for each gene were outputted from the CLC Genomic Workbench for statistical analysis in DESeq2. Under all of the diverse test conditions, the annotated coding regions showing at least one read was sufficiently deep to analyze the majority of annotated transcripts. Data were normalized with DESeq2 and the default parameters. Genes were identified as differentially expressed if their adjusted P value was <0.05 to minimize artifacts associated with multiple-comparison testing according to the Benjamini and Hochberg (BH) procedure, followed by Tukey’s multiple range test [43]. Among *E*. *histolytica* clinical strains, principal component analysis (PCA) was performed to explore the relation to the gene expression pattern. Hierarchical clustering was performed using the TCC-GUI online graphical interface [44]. Heat maps and volcano plots displaying the ˗log 10 of the p values for whole gene expression were created using the CLC Genomic Workbench. To detect the candidate function in DEGs, gene set enrichment analysis in GO term analysis and protein class identification were performed using the tools provided with PANTHER [45]. This analysis tool was used to perform the enrichment test by taking a list of genes, with each gene having a numerical value, and optimally this list is genome wide (i.e., there is a value for as many genes in a genome as possible). This tool then finds functional classes for which the genes of that class have values that are non-randomly selected from the genome-wide distribution of values. We can view uploaded data by the presence/absence of each gene following the overrepresentation test. To construct Venn diagrams, Venny (http://bioinfogp.cnb.csic.es/tools/venny/index.html) was used.

### Statistical analysis

Differences in virulence among *E*. *histolytica* clinical strains in the animal experiments were determined using the Chi-square test and Pearson’s correlation coefficient. Statistical analyses were conducted using GraphPad Prism (GraphPad Software, La Jolla, CA, USA).

## Supporting information

S1 Data Sequence information for the PCR product amplified from the D-A locus of strain Ax11. (DOCX)

S2 Data RNA-seq gene count datasets comparing two *E*. *histolytica* clinical strains. (XLSX)

S3 Data Lists of differentially expressed genes in each *E*. *histolytica* clinical strain. (XLSX)

S4 Data Lists of annotated genes and their predicted functions in the differentially expressed genes in each *E*. *histolytica* clinical strain. (XLSX)

S5 Data Distribution of GO functional classifications among the 91 strain-specific DEGs identified for the Ax19 strain. (XLSX)

S6 Data Distribution of GO functional classifications among the 49 strain-specific DEGs identified for the Ax11 strain. (XLSX)

S7 Data Distribution of GO functional classifications among the 35 strain-specific DEGs identified for the Ax22 strain. (XLSX)

S8 Data Distribution of GO functional classifications of 15 multi-functional genes among the 26 DEGs that are inversely up- or down-regulated between strains Ax19 and Ax11. (XLSX)

S9 Data Gene lists and distribution of GO functional classification of the five overlapping genes between the 91 Ax19 strain-specific DEGs and the 85 Ax19 environment-specific DEGs. (XLSX)

S1 Fig Transcriptome profiling of RNA-seq reads resulted from the HM-1 analysis in axenic and monoxenic conditions to investigate the impact of *C*. *fasciculata* on the gene expression of *E*. *histolytica*. (A) Principal component analysis of the RNA-seq reads. (B) Volcano plot showing DEGs of the HM-1 between axenic and monoxenic condition. (C) Heat map showing the clustering of each condition.
(PPTX)

## References

[1] Lozano R, Naghavi M, Foreman K, Lim S, Shibuya K, Aboyans V, et al. Global and regional mortality from 235 causes of death for 20 age groups in 1990 and 2010: a systematic analysis for the Global Burden of Disease Study 2010. Lancet. 2012;380(9859):2095–128. doi: 10.1016/S0140-6736(12)61728-0 23245604 PMC10790329

[2] Haque R, Huston CD, Hughes M, Houpt E, Petri WA Jr. Amebiasis. N Engl J Med. 2003;348(16):1565–73. doi: 10.1056/NEJMra022710 12700377

[3] Stanley SL Jr. Amoebiasis. Lancet. 2003;361(9362):1025–34. doi: 10.1016/S0140-6736(03)12830-9 12660071

[4] Marie C, Petri WA Jr. Regulation of virulence of Entamoeba histolytica. Annu Rev Microbiol. 2014;68:493–520. doi: 10.1146/annurev-micro-091313-103550 25002094 PMC9006484

[5] Loftus B, Anderson I, Davies R, Alsmark UC, Samuelson J, Amedeo P, et al. The genome of the protist parasite Entamoeba histolytica. Nature. 2005;433(7028):865–8. doi: 10.1038/nature03291 15729342

[6] Naiyer S, Kaur D, Ahamad J, Singh SS, Singh YP, Thakur V, et al. Transcriptomic analysis reveals novel downstream regulatory motifs and highly transcribed virulence factor genes of Entamoeba histolytica. BMC Genomics. 2019;20(1):206. doi: 10.1186/s12864-019-5570-z 30866809 PMC6416950

[7] Hackney JA, Ehrenkaufer GM, Singh U. Identification of putative transcriptional regulatory networks in Entamoeba histolytica using Bayesian inference. Nucleic Acids Res. 2007;35(7):2141–52. doi: 10.1093/nar/gkm028 17355990 PMC1874630

[8] MacFarlane RC, Singh U. Identification of differentially expressed genes in virulent and nonvirulent Entamoeba species: potential implications for amebic pathogenesis. Infect Immun. 2006;74(1):340–51. doi: 10.1128/IAI.74.1.340-351.2006 16368989 PMC1346599

[9] Davis PH, Zhang X, Guo J, Townsend RR, Stanley SL Jr. Comparative proteomic analysis of two Entamoeba histolytica strains with different virulence phenotypes identifies peroxiredoxin as an important component of amoebic virulence. Mol Microbiol. 2006;61(6):1523–32. doi: 10.1111/j.1365-2958.2006.05344.x 16968225

[10] Davis PH, Schulze J, Stanley SL Jr. Transcriptomic comparison of two Entamoeba histolytica strains with defined virulence phenotypes identifies new virulence factor candidates and key differences in the expression patterns of cysteine proteases, lectin light chains, and calmodulin. Mol Biochem Parasitol. 2007;151(1):118–28. doi: 10.1016/j.molbiopara.2006.10.014 17141337

[11] Escueta-de Cadiz A, Kobayashi S, Takeuchi T, Tachibana H, Nozaki T. Identification of an avirulent Entamoeba histolytica strain with unique tRNA-linked short tandem repeat markers. Parasitol Int. 2010;59(1):75–81. doi: 10.1016/j.parint.2009.10.010 19895903

[12] Singh A, Banerjee T, Kumar R, Shukla SK. Prevalence of cases of amebic liver abscess in a tertiary care centre in India: A study on risk factors, associated microflora and strain variation of Entamoeba histolytica. PLoS One. 2019;14(4):e0214880. doi: 10.1371/journal.pone.0214880 30943253 PMC6447230

[13] Ali IK, Mondal U, Roy S, Haque R, Petri WA Jr., Clark CG. Evidence for a link between parasite genotype and outcome of infection with Entamoeba histolytica. J Clin Microbiol. 2007;45(2):285–9. doi: 10.1128/JCM.01335-06 17122021 PMC1829016

[14] Ali IK, Haque R, Alam F, Kabir M, Siddique A, Petri WA Jr. Evidence for a link between locus R-R sequence type and outcome of infection with Entamoeba histolytica. Clin Microbiol Infect. 2012;18(7):E235–7. doi: 10.1111/j.1469-0691.2012.03826.x 22448930 PMC3377832

[15] Kawano-Sugaya T, Izumiyama S, Yanagawa Y, Saito-Nakano Y, Watanabe K, Kobayashi S, et al. Near-chromosome level genome assembly reveals ploidy diversity and plasticity in the intestinal protozoan parasite Entamoeba histolytica. BMC Genomics. 2020;21(1):813. doi: 10.1186/s12864-020-07167-9 33225881 PMC7681961

[16] Poole LB, Chae HZ, Flores BM, Reed SL, Rhee SG, Torian BE. Peroxidase activity of a TSA-like antioxidant protein from a pathogenic amoeba. Free Radic Biol Med. 1997;23(6):955–9. doi: 10.1016/s0891-5849(97)00066-x 9378375

[17] Diamond LS, Mattern CF, Bartgis IL. Viruses of Entamoeba histolytica. I. Identification of transmissible virus-like agents. J Virol. 1972;9(2):326–41. doi: 10.1128/JVI.9.2.326-341.1972 4335522 PMC356300

[18] Naiyer S, Bhattacharya A, Bhattacharya S. Advances in Entamoeba histolytica Biology Through Transcriptomic Analysis. Front Microbiol. 2019;10:1921. doi: 10.3389/fmicb.2019.01921 31481949 PMC6710346

[19] Watanabe K, Petri WA Jr. Molecular biology research to benefit patients with Entamoeba histolytica infection. Mol Microbiol. 2015;98(2):208–17. doi: 10.1111/mmi.13131 26173474

[20] Gilchrist CA, Petri WA. Using differential gene expression to study Entamoeba histolytica pathogenesis. Trends Parasitol. 2009;25(3): 124–31. doi: 10.1016/j.pt.2008.12.007 19217826 PMC2930180

[21] Pacheco-Yepez J, Jarillo-Luna RA, Gutierrez-Meza M, Abarca-Rojano E, Larsen BA, Campos-Rodriguez R. Peroxynitrite and peroxiredoxin in the pathogenesis of experimental amebic liver abscess. Biomed Res Int. 2014;2014: 324230. doi: 10.1155/2014/324230 24822193 PMC4009108

[22] Konig C, Honecker B, Wilson IW, Weedall GD, Hall N, Roeder T, et al. Taxon-specific proteins of the pathogenic Entamoeba species E. histolytica and E. nuttalli. Front Cell Infect Microbiol. 2021;11: 641472. doi: 10.3389/fcimb.2021.641472 33816346 PMC8017271

[23] Brachhaus I, Roeder T, Lotter H, Schwerdtfeger M, Tannich E. Differential gene expression in Entamoeba histolytica isolated from amoebic liver abscess. Mol Microbiol. 2002;44(4): 1063–72. doi: 10.1046/j.1365-2958.2002.02941.x 12010498

[24] Weedall GD, Sherrington J, Paterson S, Hall N. Evidence of gene conversion in genes encoding the Gal/GalNac lectin complex of Entamoeba. PLoS Negl Trop Dis. 2011;5(6): e1209. doi: 10.1371/journal.pntd.0001209 21738808 PMC3125142

[25] Teixeira JE, Huston CD. Participation of the serine-rich Entamoeba histolytica protein in amebic phagocytosis of apoptotic host cells. Infect Immun. 2008;76(3): 959–66. doi: 10.1128/IAI.01455-07 18086807 PMC2258814

[26] Khalil MI, Foda BM, Suresh S, Singh U. Technical advances in trigger-induced RNA interference gene silencing in the parasite Entamoeba histolytica. Int J Parasitol. 2016;46(3): 205–12. doi: 10.1016/j.ijpara.2015.11.004 26747561 PMC4767557

[27] Kangussu-Marcolino MM, Morgado P, Manna D, Yee H, Singh U. Development of a CRISPR/Cas9 system in Entamoeba histolytica: proof of concept. Int J Parasitol. 2021;51(2–3): 193–200. doi: 10.1016/j.ijpara.2020.09.005 33264648 PMC7880892

[28] Nakada-Tsukui K, Sekizuka T, Sato-Ebine E, Escueta-de Cadiz A, Ji DD, Tomii K, et al. AIG1 affects in vitro and in vivo virulence in clinical isolates of Entamoeba histolytica. PLoS Pathog. 2018;14(3):e1006882. doi: 10.1371/journal.ppat.1006882 29554130 PMC5884625

[29] Davis PH, Schulze J, Stanley SL. Transcriptomic comparison of two Entamoeba histlytica strains with defined virulence phenotypes identifies new virulence factor candidates and key differences in the expression patterns of cysteine proteases, lectin light chains, and calmodulin. Mol Biochem Parasitol. 2007;151(1): 118–28. 17141337 10.1016/j.molbiopara.2006.10.014

[30] Nagaraja S, Cai MW, Sun J, Varet H, Sarid L, Trebicz-Geffen M, et al. Queuine Is a Nutritional Regulator of Entamoeba histolytica Response to Oxidative Stress and a Virulence Attenuator. mBio. 2021;12(2). doi: 10.1128/mBio.03549-20 33688012 PMC8092309

[31] Hertz R, Tovy A, Kirschenbaum M, Geffen M, Nozaki T, Adir N, et al. The Entamoeba histolytica Dnmt2 homolog (Ehmeth) confers resistance to nitrosative stress. Eukaryot Cell. 2014;13(4):494–503. doi: 10.1128/EC.00031-14 24562908 PMC4000097

[32] Clark CG, Diamond LS. Methods for cultivation of luminal parasitic protists of clinical importance. Clin Microbiol Rev. 2002;15(3):329–41. doi: 10.1128/CMR.15.3.329-341.2002 12097242 PMC118080

[33] Robinson GL. The Laboratory Diagnosis of Human Parasitic Amoebae. Transactions of the Royal Society of Tropical Medicine and Hygiene. 1968;62(2):285–94. doi: 10.1016/0035-9203(68)90170-3 4296113

[34] Kobayashi S, Imai E, Haghighi A, Khalifa SA, Tachibana H, Takeuchi T. Axenic cultivation of Entamoeba dispar in newly designed yeast extract-iron-gluconic acid-dihydroxyacetone-serum medium. J Parasitol. 2005;91(1):1–4. doi: 10.1645/GE-3386 15856863

[35] Suzuki J, Kobayashi S, Murata R, Tajima H, Hashizaki F, Yanagawa Y, et al. A survey of amoebic infections and differentiation of an Entamoeba histolytica-like variant (JSK2004) in nonhuman primates by a multiplex polymerase chain reaction. J Zoo Wildl Med. 2008;39(3):370–9. doi: 10.1638/2007-0171.1 18816999

[36] Rigothier MC, Khun H, Tavares P, Cardona A, Huerre M, Guillen N. Fate of Entamoeba histolytica during establishment of amoebic liver abscess analyzed by quantitative radioimaging and histology. Infect Immun. 2002;70(6):3208–15. doi: 10.1128/IAI.70.6.3208-3215.2002 12011016 PMC128000

[37] Diamond LS, Harlow DR, Cunnick CC. A new medium for the axenic cultivation of Entamoeba histolytica and other Entamoeba. Trans R Soc Trop Med Hyg. 1978;72(4):431–2. doi: 10.1016/0035-9203(78)90144-x 212851

[38] Royer TL, Gilchrist C, Kabir M, Arju T, Ralston KS, Haque R, et al. Entamoeba bangladeshi nov. sp., Bangladesh. Emerg Infect Dis. 2012;18(9):1543–5. doi: 10.3201/eid1809.120122 22932710 PMC3437720

[39] Gotfred-Rasmussen H, Lund M, Enemark HL, Erlandsen M, Petersen E. Comparison of sensitivity and specificity of 4 methods for detection of Giardia duodenalis in feces: immunofluorescence and PCR are superior to microscopy of concentrated iodine-stained samples. Diagn Microbiol Infect Dis. 2016;84(3):187–90. doi: 10.1016/j.diagmicrobio.2015.11.005 26707069

[40] Ali IK, Zaki M, Clark CG. Use of PCR amplification of tRNA gene-linked short tandem repeats for genotyping Entamoeba histolytica. J Clin Microbiol. 2005;43(12):5842–7. doi: 10.1128/JCM.43.12.5842-5847.2005 16333065 PMC1317169

[41] Hon CC, Weber C, Sismeiro O, Proux C, Koutero M, Deloger M, et al. Quantification of stochastic noise of splicing and polyadenylation in Entamoeba histolytica. Nucleic Acids Res. 2013;41(3):1936–52. doi: 10.1093/nar/gks1271 23258700 PMC3561952

[42] Wang L, Nie J, Sicotte H, Li Y, Eckel-Passow JE, Dasari S, et al. Measure transcript integrity using RNA-seq data. BMC Bioinformatics. 2016;17:58. doi: 10.1186/s12859-016-0922-z 26842848 PMC4739097

[43] BENJAMINI Y. Controlling The False Discovery Rate—A Practical And Powerful Approach To Multiple Testing. Journal of the Royal Statistical Society. 1995;57:289–300.

[44] Su W, Sun J, Shimizu K, Kadota K. TCC-GUI: a Shiny-based application for differential expression analysis of RNA-Seq count data. BMC Res Notes. 2019;12(1):133. doi: 10.1186/s13104-019-4179-2 30867032 PMC6417217

[45] Mi H, Poudel S, Muruganujan A, Casagrande JT, Thomas PD. PANTHER version 10: expanded protein families and functions, and analysis tools. Nucleic Acids Res. 2016;44(D1):D336–42. doi: 10.1093/nar/gkv1194 26578592 PMC4702852

