# Supplementary material for: Gene expression of axenically-isolated clinical Entamoeba histolytica strains and its impact on disease severity of amebiasis
Source: PLoS Pathog. 2022 Sep 30;18(9):e1010880. doi: 10.1371/journal.ppat.1010880 (PMC9555656; doi:10.1371/journal.ppat.1010880)
Supplement: S1 Data — (DOCX) [file ppat.1010880.s001.docx]

**S1 Data. Sequence information for the PCR product amplified from the DA locus of strain Ax 11**

TGAGTTCTCNNTTTATACTTTTATATGTTTATATGTTTATATCCTTATTTATTATTCTTTTATATTCTTATCACTTCCTACTACTCTTATTTATTATCCTTATTATATCTATTCTTACTCCCTATCTTTATTATCTTTATTATCTTTATTACCTTTATTACCTTTATTACCTTTATTACCTTTATTACCTTTATTACCTTTATTACTTTTATTACTTTTATTATCTTTATTACCTTTATTATATCTATTCTCACTTCCTATACGTACTCTTTTTACTACTCTTCTTACTATACCTCTTACTACTCCTACTTTCACCTCCCTCTTTAATTGTTAAN

Repeated sequences

CTTTATTAT

CTTTATTAC

TTTTATTAC

TTTTATTAT

CTTTTTACTACT
