# Supplementary material for: Gene expression of axenically-isolated clinical Entamoeba histolytica strains and its impact on disease severity of amebiasis
Source: PLoS Pathog. 2022 Sep 30;18(9):e1010880. doi: 10.1371/journal.ppat.1010880 (PMC9555656; doi:10.1371/journal.ppat.1010880)
Supplement: S1 Fig — (A) Principal component analysis of the RNA-seq reads. (B) Volcano plot showing DEGs of the HM-1 between axenic and monoxenic condition. (C) Heat map showing the clustering of each condition. (PPTX) [file ppat.1010880.s010.pptx]

## Slide 1
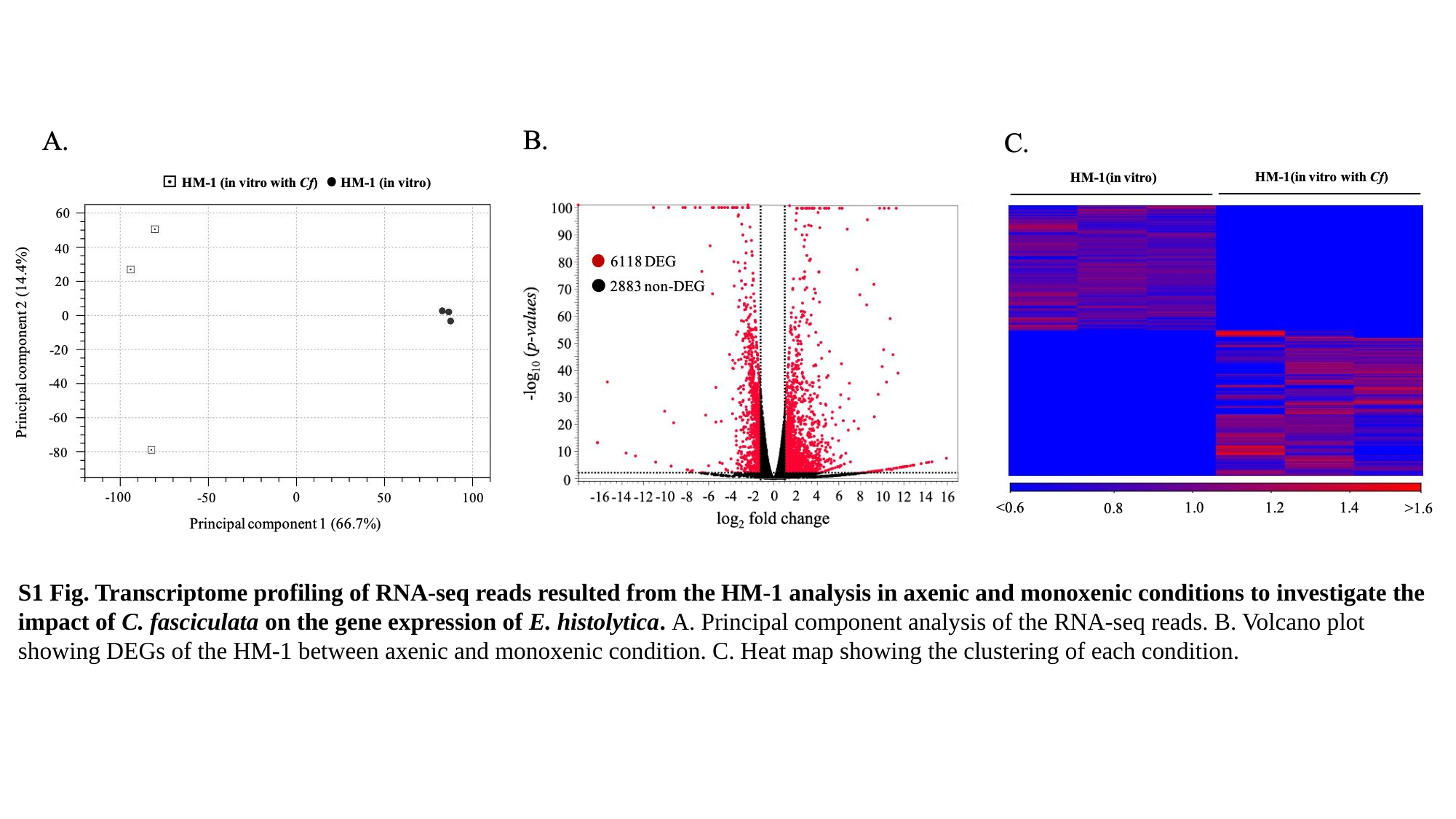

S1 Fig. Transcriptome profiling of RNA-seq reads resulted from the HM-1 analysis in axenic and monoxenic conditions to investigate the impact of C. fasciculata on the gene expression of E. histolytica. A. Principal component analysis of the RNA-seq reads. B. Volcano plot showing DEGs of the HM-1 between axenic and monoxenic condition. C. Heat map showing the clustering of each condition.
